# Supplementary material for: Chromothripsis during telomere crisis is independent of NHEJ, and consistent with a replicative origin
Source: Genome Res. 2019 May;29(5):737–49. doi: 10.1101/gr.240705.118 (PMC6499312; doi:10.1101/gr.240705.118)
Supplement: Supplemental Material [file supp_gr.240705.118_Supplemental_file_1.zip › contigs/annotated_contigs/DB81/contig.2.DB81_length_701_mean_cov_7.7032810271.docx]

**DB81_length_701_mean_cov_7.7032810271**

```
AGACTTTGGGTCACATGCACACTCATGAACAAATCACTGTGGACAGGCGGCAGGTAGAGTGTGGTGATTGGATTATCTTAGATTACATG
 >chr6:14312446-14312759 + E=1e-176 p=3e-03
CTTCACTCTCAAGCCTGGGGGATGGAGTTGGCTGAATGAAAACAATATAGACTAAGAGTTGGTGGATATACGCCCAATAATATTAGGGC

ACTGCTGCCTCTAGATGGGAGAGTGGAGAGTGAGTGGTATAAACAACAAATATCCGCTAAACTACCGCAGAAAAAGGAGCAATAATTGA

AAAAAGTAAAGCACTTTGAATCTCTTGAATTAAAAAGCAATAT|GAA|ATAACTGGCATCCCAAACCCTGAATTCAGTCCTGCCAGCTC
                                            >chr6:14314594-14314985 + E=2e-223
TAGGTGACTACCTTGGCCTGCTTGGACTGCTATAATGGATACCATAGACTGGGTGGCTTAAACAATAAACATATATTTCACACAGTTCT

AGAGGCTGGGAAGTCCAAGATCTAGGTGCTGACAGATCTGGTGTCTGGTGAGGGCTCCTCAGTTCCCATTTGCAAACATTTGGTCCATA

TGGACAAACACTTAGTCCATAACAGTTACTAAGAGAACTGACATTTCCTGAGGACCTATTAGTTGCCGCACAAGTTATTTATGTCATCT

CATTTAATCATCACAGCAGGCCTAATGAAGTGGGAACTGTTTTTCCAATTTTATACATGAGGAAACTGAGGAACGACTAG
```
